# Reporte y mapeo de megacolon en una población colombiana: análisis de 10 años y 937 casos

**DOI:** 10.15446/rsap.V25n4.105243

**Published:** 2023-07-01

**Authors:** Jhony A. Díaz-Vallejo, Ivan D. Lozada-Martinez, Cristian D. Benavides-Molina, Henry E. Sánchez-Mesa, Leonardo F. Gil-Montoya

**Affiliations:** 1 JD: MD. Facultad de Ciencias para la Salud, Universidad de Caldas. Manizales, Colombia. Semillero de Investigación en Cirugía Pediátrica, Universidad de Caldas. Grupo de Investigación en Nutrición, Metabolismo y Seguridad Alimentaria, Universidad de Caldas. Manizales, Colombia Alejandrodiazval@gmail.com Universidad de Caldas Grupo de Investigación en Nutrición, Metabolismo y Seguridad Alimentaria Universidad de Caldas Manizales Colombia; 2 IL: MD. Universidad de la Costa. Barranquilla, Colombia. ilozada@cuc.edu.co Universidad de la Costa Universidad de la Costa Barranquilla Colombia ilozada@cuc.edu.co; 3 CB: MD. Facultad de Ciencias para la Salud, Universidad de Caldas. Manizales, Colombia. Semillero de Investigación en Cirugía Pediátrica, Universidad de Caldas. Manizales, Colombia. Cristiandavidbm96@gmail.com Universidad de Caldas Semillero de Investigación en Cirugía Pediátrica Universidad de Caldas Manizales Colombia; 4 HS: MD. Facultad de Ciencias para la Salud, Universidad de Caldas. Manizales, Colombia. henry_sa19@hotmail.com Universidad de Caldas Facultad de Ciencias para la Salud Universidad de Caldas Manizales Colombia; 5 LG: MD. Ped. Universidad de Caldas. Manizales, Colombia. Semillero de Investigación en Cirugía Pediátrica, Universidad de Caldas. Manizales, Colombia. leogm12@gmail.com Universidad de Caldas Semillero de Investigación en Cirugía Pediátrica Universidad de Caldas Manizales Colombia

**Keywords:** Megacolon, enfermedad de Hirschsprung, mapeo geográfico, epidemiología, Colombia *(fuente: DeCS, BIREME)*, Megacolon, hirschsprung disease, geographic mapping, epidemiology, Colombia *(source: MeSH, NLM)*

## Abstract

**Objetivo:**

Analizar el reporte epidemiológico y el comportamiento geoespacial del megacolon en el departamento de Caldas.

**Métodos:**

Estudio retrospectivo de corte transversal, donde se analizaron datos de la territorial de salud regional de Caldas, compatibles con sospecha o diagnóstico definitivo de enfermedad de Hirschprung y megacolon durante 2009-2019. Se realizó un análisis univariado para el cálculo de frecuencias y porcentajes. Se utilizó QGIS v.3.24 para establecer la localización geoespacial de los casos.

**Resultados:**

Se reportaron 937 casos en total (55% clasificados como megacolon, con código CIE-10 K593). El 2013 y el 2009, fueron los años con mayor y menor número de casos reportados (145 vs. 40 casos). El 70% de los casos fueron de impresión diagnóstica (n=652) y solo el 10% correspondió a definitivos (N=98). El 75,7% se presentó en menores de edad y el 85% (n=782) correspondió a casos propios del departamento de Caldas, principalmente en Manizales (n=643).

**Conclusiones:**

Entre el 2009 y el 2019 se evidenció en el departamento de Caldas, una prevalencia elevada de casos de megacolon, con una tendencia reducida en los últimos años. Siete de cada 10 casos se reporta como impresión diagnóstica, y en esta misma proporción se presentó en menores de edad. Solo el 15% de los casos proviene de fuera del departamento. Finalmente, se observó que la base disponible es limitada con respecto a datos sociodemográficos o clínicos que permitan analizar de manera más profunda una enfermedad que no posee datos epidemiológicos sólidos.

## Abstract (en)

**Objetive:**

To analyze the epidemiological report and geospatial behavior of megacolon in the department of Caldas.

**Methods:**

Retrospective cross-sectional study, where data from the Caldas regional health territorial, compatible with suspicion or definitive diagnosis of Hirschprung's disease and megacolon during 2009-2019 were analyzed. Univariate analysis was per-formed to calculate frequencies and percentages. QGIS v.3.24 was used to establish the geospatial location of the cases.

**Results:**

A total of 937 cases were reported (55% classified as megacolon, with ICD-10 code K593). The years 2013 and 2009 were the years with the highest and lowest number of cases reported (145 vs. 40 cases). Seventy percent of the cases were of diagnostic impression (n=652) and only 10% corresponded to definitive cases (n=98). The 75.7% occurred in minors and 85% (n=782) corresponded to cases in the department of Caldas, mainly in Manizales (n=643).

**Conclusions:**

Between 2009 and 2019, a high prevalence of megacolon cases was evidenced in the department of Caldas, with a reduced trend in recent years. 7 out of 10 cases are reported as diagnostic impression and in this same proportion, it occurred in minors. Only 15% of the cases come from outside the department. Finally, it was observed that the available data base is limited with respect to sociodemographic or clinical data, which would allow a more in-depth analysis of a disease that does not have solid epidemiological data.

Cuando se habla de megacolon, se conocen dos condiciones adicionales a la enfermedad de Hirschsprung: megacolon idiopático y megacolon adquirido 1. El mega colon idiopático es una condición que implica la dilatación persistente del colon en ausencia de enfermedad orgánica, también llamada megacolon funcional, psicógeno, megarrecto, inercia colónica o rectal, o simplemente estreñimiento crónico 2. El megacolon adquirido se desarrolla en pacientes con un trastorno causal demostrable, como, por ejemplo, obstrucción anorrectal, uso de medicamentos antiespasmódicos, infecciones intestinales, enfermedad de Chagas, enterocolitis necrosantes, enfermedad inflamatoria intestinal, afecciones metabólicas o autoinmunes y trastornos del sistema nervioso central o endocrino 3.

Alrededor del mundo, se estima una incidencia de la enfermedad de aproximadamente 1 en cada 5 000 nacidos vivos, siendo cuatro veces más común en varones que en hembras 1. Aunque existe poca evidencia, se estima que a lo largo del tiempo la enfermedad tiende a progresar, afectando considerablemente la capacidad funcional, la calidad de vida y la integridad física del afectado 4,5. En la edad adulta se ha descrito que entre los síntomas más comunes se encuentran el estreñimiento, la distensión, el malestar por gases y el dolor abdominal dolor; en los niños se manifiesta principalmente con impactación fecal 5. Adicionalmente, se ha asociado con condiciones neuropsiquiátricas que incluyen esquizofrenia y déficit cognitivo, así como condiciones neurológicas agudas como enfermedad cerebrovascular y epilepsia. Considerando la heterogeneidad de las manifestaciones y el sinnúmero de posibles diagnósticos, pueden existir retrasos y dificultades en el manejo definitivo, el cual es quirúrgico 3.

En Colombia no existen datos relacionados sobre el comportamiento epidemiológico, el manejo o los desenlaces del megacolon, a pesar de ser una entidad patológica de interés en cirugía pediátrica o del adulto. Esto demuestra la inexistencia de hojas de ruta o protocolos específicos de manejo adaptados a los contextos poblacionales de las distintas regiones. De acuerdo a las recomendaciones técnicas en investigación e innovación del Instituto Nacional de Salud 6 sobre prioridades de investigación en salud pública, se encuentra el diseño y el registro de datos fidedignos regionales que permitan conocer la morbilidad regional y nacional, y sobre ello ejecutar programas para controlar la carga de enfermedades o complicaciones prevenibles 6. Esto es más intenso al relacionarlo con las prioridades por enfoque territorial, donde se debe indagar sobre la desigualdad de morbilidad en poblaciones más vulnerables, debido a dificultades en el acceso oportuno a servicios de salud básicos y especializados 7. Estos documentos técnicos gubernamentales muestran la morbilidad distribuida por grandes causas, pero las enfermedades quirúrgicas no se discriminan en estos resultados 6,7, lo cual obliga a la búsqueda y el análisis de datos de interés en salud pública de forma independiente, que sirvan de base para el diseño de políticas públicas y estrategias dirigidas al control de la carga de enfermedad específica. Las territoriales distritales y departamentales reciben datos desde las instituciones prestadoras de servicios de salud o las entidades promotoras de salud, pero no siempre son analizadas y publicadas. Con base en lo anterior, el objetivo de este estudio fue analizar el reporte epidemiológico y el comportamiento geoespacial del megacolon en el departamento de Caldas.

## MATERIALES Y MÉTODOS

### Diseño del estudio

Estudio retrospectivo de corte transversal que incluyó a personas con impresión diagnóstica, diagnóstico confirmado o antecedente de enfermedad de Hirschprung y megacolon, con los códigos CIE-10 Q431 y K593, respectivamente, en el departamento de Caldas, Colombia, durante los años 2009 a 2019. El departamento de Caldas se encuentra ubicado en el Eje Cafetero de Colombia, con una población para 2019 de 998 255 habitantes.

### Variables evaluadas y extracción de datos

La información fue extraída de una base de datos suministrada por la territorial del departamento de Caldas, sirviendo como unidad de análisis. Se organizó y depuró la base de datos y se seleccionaron las variables epidemiológicas disponibles. Las variables evaluadas fueron: edad, zona de procedencia, enfermedad y tipo de diagnóstico.

### Análisis de los datos

Para el análisis de los datos, estos se organizaron en una matriz de Excel y posteriormente en el programa SPSS versión 25, licenciado para la Universidad de Caldas, y se analizaron mediante estadística descriptiva. Para ubicar el lugar de residencia de los casos, se hizo una localización geoespacial de estos con el programa QGIS v.3.24.

### Declaraciones éticas

Este estudio no necesitó aprobación por parte de comité de ética, teniendo en cuenta que se utilizaron datos anónimos y públicos. Cumplió con la Declaración de Helsinki 8 y fue clasificado en la categoría de investigación sin riesgo, según el artículo 11 de la Resolución 8430 de 1993 del Ministerio de Salud de Colombia 9.

## RESULTADOS

**Figura 1:**
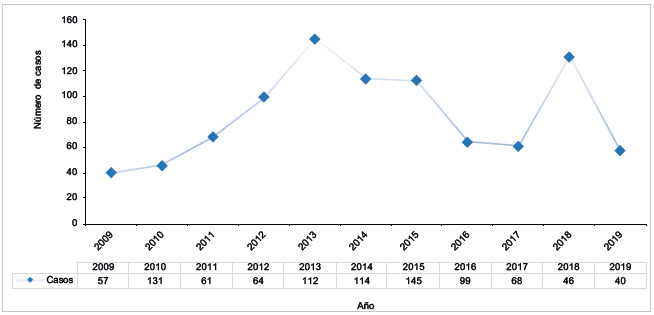
Frecuencia de casos en Caldas entre 2009-2019

Se obtuvo un total de 937 casos durante las consultas y hospitalizaciones entre 2009 y 2019 para el departamento de Caldas. Durante este periodo, se identificó que el año con mayor número de casos fue el 2013 con 145 casos, frente al 2009, en el cual se reportó un menor número de casos (n=40) (Figura 1). Hubo en total 652 (70%) casos de impresión diagnóstica, 98 (10%) casos confirmados y 187 (20%) casos con antecedente de Hirschprung o megacolon. 

**Figura 2:**
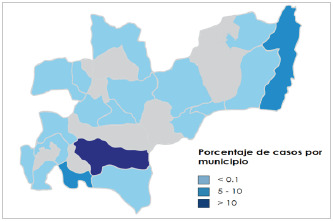
Distribución del porcentaje de casos de megacolon en el departamento de caldas, entre 2009 y 2019

Se encontraron 520 (55,5%) casos clasificados con el código CIE-10 K593 (megacolon) y 417 (44,5%) con el código Q431 (enfermedad de Hirschprung). En la distribución según edad, se encontró una mayor frecuencia de casos para los infantes (< 18 años), tanto para los diagnósticos de Hirschprung como para los de megacolon, con 710 (75,7%) casos de infantes frente a 227 (24,3%) casos para mayores de edad. El 83,4% (n=782) de los pacientes correspondió a pacientes propios del departamento de Caldas, en tanto que el otro 15% correspondió a pacientes provenientes de distintas zonas del país (Tabla 1). Al identificar esta distribución en los municipios del departamento de Caldas, se encontró que de los 782 casos, 643 provinieron de la ciudad de Manizales, seguido de los municipios de Chinchiná y La Dorada, con 61 y 21 casos, respectivamente (Figura 2). 

Se encontraron 520 (55,5%) casos clasificados con el código CIE-10 K593 (megacolon) y 417 (44,5%) con el código Q431 (enfermedad de Hirschprung). En la distribución según edad, se encontró una mayor frecuencia de casos para los infantes (< 18 años), tanto para los diagnósticos de Hirschprung como para los de megacolon, con 710 (75,7%) casos de infantes frente a 227 (24,3%) casos para mayores de edad. El 83,4% (n=782) de los pacientes correspondió a pacientes propios del departamento de Caldas, en tanto que el otro 15% correspondió a pacientes provenientes de distintas zonas del país (Tabla 1). Al identificar esta distribución en los municipios del departamento de Caldas, se encontró que de los 782 casos, 643 provinieron de la ciudad de Manizales, seguido de los municipios de Chinchiná y La Dorada, con 61 y 21 casos, respectivamente (Figura 2).

**Tabla 1:** Lugar de residencia de los casos de megacolon atendidos en el departamento de caldas

Departamento	Frecuencia	Porcentaje
Caldas	782	83,4%
Tolima	58	6,18%
Cundinamarca	44	4,7%
Antioquia	19	2,02%
Risaralda	15	1,6%
Otro	11	1,17%
Guajira	4	0,42%
Valle del cauca	3	0,32%
Nariño	1	0,19%
Total	937	100%

## DISCUSIÓN

En los últimos años se ha reportado un incremento notable en la prevalencia de megacolon en comparación con décadas anteriores 10. Sin embargo, en este estudio solo 98 de los casos fueron reportados con diagnóstico definitivo, donde se presume que este se realizó con el soporte de análisis histopatológico, el cual es un criterio necesario para establecer el diagnóstico final. Sin embargo, esto no se puede corroborar en las bases estadísticas de dominio público, al no existir una variable explicita. El alto número de casos sospechados evidenciados puede explicarse por la conducta preventiva de los médicos tratantes, ante posibles complicaciones asociadas al megacolon, teniendo en cuenta que, por lo menos en neonatos o lactantes menores, se puede evitar potencialmente el desarrollo de enterocolitis, síndrome de tapón de meconio asociado y la perforación colónica 11,12.

Para el código de diagnóstico por registrar en la historia clínica, existen dos, el megacolon (K593) y la enfermedad de Hirschsprung (Q431), a pesar de no existir diferencias significativas en los protocolos de ruta de manejo. El 55,5% de los casos reportados en el periodo estudiado en el departamento de Caldas se hizo con el diagnóstico de megacolon. Entonces, es notable que se utilizan ambos diagnósticos de manera frecuente para la sospecha de esta condición patológica. Este comportamiento puede explicarse porque algunos médicos prefieren, en su ejercicio médico, evitar el uso de epónimos en sus diagnósticos, por la discusión actual que se tiene con el uso de estos 13. Otros, por el hecho de no estar seguros del diagnóstico sospechado, ya sea por la edad, o por las diferencias precisas entre estas dos condiciones que comparten características clínicas muy similares, razón por la cual se decidió incluir ambos diagnósticos para el presente trabajo.

Si bien la enfermedad de Hirschsprung se diagnostica mayormente en infantes, también se debe considerar en el adolescente, el joven o el adulto, por variantes de inicio tardío, toda vez que esta entidad hace referencia a la afección de 2 o 3 cm distales del recto, cuya manifestación puede causar síntomas durante varios años de severa constipación o impactación fecal. Por eso, se estima que solamente el 10% de los pacientes con enfermedad de Hirschsprung son adultos 14. En nuestro análisis, se observaron 227 casos en personas mayores de 18 casos. No obstante, ello pudo deberse a los casos establecidos como antecedentes, lo que afecta la precisión de la estimación.

Según nuestro conocimiento, este sería el primer estudio que se hace en Caldas y Colombia con el objetivo de conocer el comportamiento y la distribución de esta enfermedad. Se notó una persistencia subjetiva de casos a través del tiempo en ciertas zonas del departamento, como lo son el Magdalena y el norte caldense. La heredabilidad en esta patología se ha identificado en más del 80% de los casos, encontrándose asociaciones con el sistema nervioso entérico, así como síndromes monogénicos y cromosómicos como el síndrome de Down 15; por ello, es imperativo diseñar y ejecutar estudios más detallados sobre esta condición, tanto en el departamento de Caldas como en Colombia, para conocer la ecología genética y epigenética relacionada con el comportamiento epidemiológico de esta enfermedad.

Entre las limitaciones encontradas, es necesario manifestar que los datos disponibles y reportados a las territoriales departamentales de salud deben tener un mayor número de variables que permitan el análisis de condiciones que no son abordadas en los documentos técnicos nacionales y publicados por el Instituto Nacional de Salud o el Ministerio de Salud y Protección Social de Colombia. De esta forma, se puede conocer a profundidad enfermedades que tienen un comportamiento o manifestación más agresiva en zonas o regiones particulares del país, y diseñar estrategias basadas en la evidencia, soportadas con datos masivos y fidedignos. Lo anterior, debido a que en la base consultada no existían datos distintos a los analizados, por lo que nuestras conclusiones se circunscriben netamente a la distribución de casos por municipios, por edad y tipo de diagnóstico. Entonces, no es posible plantear hipótesis más complejas que ayuden a comprender este fenómeno. Adicionalmente, se debe resaltar que los códigos diagnósticos CID-10 para patologías relacionadas con enfermedad de Hirschprung no siempre son únicos, por lo que se pudieron omitir más casos al tomar más códigos. Es posible que los casos identificados en Manizales no sean propios de la ciudad, pero tuvieron el diagnóstico allí, lo que puede sesgar el resultado obtenido.

Entre el 2009 y el 2019 se evidenció en el departamento de Caldas un incidencia elevada de casos de megacolon, con una tendencia reducida en los últimos años. Siete de cada 10 casos se reportan como impresión diagnóstica, y en esta misma proporción se presentó en menores de edad. Solo el 15% de los casos proviene de fuera del departamento. Finalmente, se observó que la base disponible es limitada con respecto a datos socio-demográficos o clínicos que permitan analizar de manera más profunda una enfermedad que no tiene datos epidemiológicos sólidos ♦
